# A case study: lessons learned from online tutorial to improve practice readiness for family medicine residents in Palestine

**DOI:** 10.1186/s12909-024-05163-1

**Published:** 2024-03-08

**Authors:** Benjamin Colton, Lubna Saudi, Ann Smalldridge, Nicki Spicer, Therese Zink

**Affiliations:** 1 Hashemite University, Zarqa, Jordan; 2 Division and Residency Program, An-Najah National University, Nablus, Palestine; 3 Manchester, UK; 4 South Devon, UK; 5 Warren Alpert Medical School, Brown University, Rhode Island, USA

**Keywords:** Family Medicine, Online tutorial, Virtual supervision, Cross cultural training, Developing countries, Middle/low-income countries, Palestine

## Abstract

**Background:**

Geopolitical and socioeconomic challenges limit faculty development and clinical teaching in Palestine and many other developing countries. The first, and still only, Family Medicine (FM) residency program is a four-year program based out of An-Najah University in the West Bank. Training in primary care clinics occurs in the final two years and there are many challenges to adequate supervision in the clinical setting that were exacerbated during the pandemic. To improve the readiness for practice skills of 13 Palestinian FM residents a three-month tutorial program was organized in 2020. A nongovernmental organization (NGO) that has worked to support Family Medicine development in the region engaged experienced British and American General Practitioners trained as tutors to offer online tutorials. We examined the program as a case study to understand the factors that facilitated or impaired a positive virtual learning environment in a middle/low income country.

**Methods:**

The tutors and residents were divided into groups and met virtually between June and September 2020. Evaluations and session reports collected during the program, the text of an online chat, and responses to an online survey two years later were collected. Using thematic analysis techniques, we evaluated the value for the residents at the time and two years later and identified factors that facilitated or impaired a positive virtual learning environment.

**Results:**

Themes of knowledge, skills, attitudes, cultural disconnects, and tutorial logistics emerged. The group with the most stable tutor pairing, including one Arabic-speaker familiar with the context, was the most engaged. The all-female group formed a chat group to share real-time case questions during clinical practice and focused on skills (e.g. conducting a thorough medication review) and attitudes (e.g. open to sharing and discussing uncertainties). Other groups were less cohesive.

**Conclusions:**

Transnational tutorials that focused on clinical thinking and decision-making skills were most successful when the tutorial pair was stable, offered familiarity with the language and addressed cultural differences. Intrinsic factors such as lacking the motivation to participate and extrinsic factors such as unstable internet and rolling electric cuts, and clinical structures that made applying new skills challenging were more difficult to address but must be considered.

**Supplementary Information:**

The online version contains supplementary material available at 10.1186/s12909-024-05163-1.

## Background

Family Medicine (FM) is a new specialty in Palestine. The first, and still only, Family Medicine Residency Program is based out of An Najah University (ANNU) in Nablus in the Northern West Bank. ANNU operates as a public university and currently has 22,000 students and 300 professors across 19 faculties, which makes it the largest university in Palestine. The Faculty of Medicine was established in 1999 after receiving accreditation by the Ministry of Education and Higher Education. The Family Medicine residency program started in 2011 at ANNU with an enrollment of five first year residents. The program currently has 46 residents across its four years of training. The residents are contracted Palestinian Ministry of Health (MOH) employees who MOH selects to participate in the residency program. During the final two years of the program, the MOH places the residents in one of their primary care clinics. These clinics are scattered throughout the country, are at a variety of locations (rural vs. urban), and have varied facilities (comprehensive with lab testing available vs. limited lab, specialists at the clinic vs. only one primary care doctor). Electronic health records are not yet universally used.

Besides one day of protected time each week reserved for an academic learning day, third- and fourth-year residents at ANNU spend the rest of their time in primary care clinics treating patients. A variety of geopolitical and socio-economic challenges limit teaching in their clinical settings. Some residents see 100–150 patients/day, as do their trainers, leaving them little potential teaching/learning time. Also, trainers are not paid extra to provide clinical supervision to residents and have little to no training about how to supervise. The lack of monetary incentive leads trainers to prioritize other responsibilities rather than teaching. The lack of training on supervision skills leaves them with little guidance on how to mentor their trainees. Furthermore, the residents are spread around the country, often at completely different locations than their supervisors/trainers, leaving the residents on their own to treat patients without adequate supervision. The COVID-19 pandemic created an increased strain on the medical system and restricted movement, further exacerbating the lack of clinical supervision. Due to this combination of problems, a group of 13 residents nearing the end of their fourth year of residency reported feeling unprepared for their upcoming examinations and future independent practice. Local residency faculty as well as visiting faculty observed a lack of training in clinical decision making skills.

The nongovernmental organization (NGO) Foundation for Family Medicine in Palestine (FFMP) was created in 2012 to support the development of the FM specialty in Palestine and made week-long trips two to three times a year to conduct training workshops for the local faculty and residents. Volunteer United Kingdom (UK) and American general practitioners (GPs) who were all family medicine physicians certified to train family medicine specialists led the trainings.

Prior to the COVID-19 pandemic, e-learning occurred around the world [1] and in Palestine [2–4]. E-learning tutorial formats were used by NGOs to enhance the education of medical students [2] and to address the ongoing learning needs of practicing physicians [3]. FFMP used it in a limited fashion with ANNU residents to supplement the skills of young faculty [4].

In order to supplement the clinical decision making training of 4th year residents in Palestine, FFMP launched a three-month program of online tutorials in June 2020. The overarching program goal was to provide focused, interactive, practical training for the residents in order to improve their clinical decision making skills in a patient-centered manner. On literature review, no studies were available from the Middle East evaluating the effectiveness of this type of training. Furthermore, no literature is available addressing the use of transnational online tutorials to supplement the clinical decision making skills of residents. Also, most studies focusing on online education in health care were performed in high-income countries. The purpose of this paper is to answer the question of which factors facilitated or impaired a positive virtual learning environment during the online practice readiness training of residents in a middle/low-income country.

## Methods

### Study design

We used a case study research design [5] to evaluate the online tutorial program.

#### Settings and participants

Thirteen family medicine residents in Palestine, nearing the end of their fourth year of residency, participated in the tutorials. Volunteer trainers from the UK and US facilitated the tutorial sessions.

The residency program director (LS) worked alongside the lead UK GP trainer (AS) to develop the goals and format for the online tutorials. The tutorial objectives are listed in Table 1. The skills and attitudes to be addressed were derived from UK tutor training materials [6]. The 13 residents were divided into three groups by LS. Recognizing the time commitment for volunteer trainers and the challenge of schedules, three tutors were allocated to each group with the plan that at least two would be available for each session. Where possible the tutors included an experienced GP trainer with prior experience teaching in the NGO’s program and a new volunteer who was being trained for future tutorials. Based on earlier experience, at least one Arabic speaker was assigned to each group [4]. Tutors were oriented to the proposed case-based discussion format and urged to cover the 12 skills and attitudes listed in Table 2 derived from UK training materials (Column 1) [6].

**Table 1:** Goals for the online tutorials outlined by UK tutors and ANNU FM residency director

• To grow their critical thinking skills and decision-making skills
• To improve their skills and confidence in managing complex conditions and long-term continuity of care
• To improve their knowledge, skills and attitudes and behaviors in self-directed learning
• To help expand their learning skills in the Art of Family Medicine approach to patient-centered care
• To prepare them for their upcoming Family Medicine Board examination
• Provide a boost in their clinical training to make up for a lack of on-the-job clinical supervision and
• To demonstrate the value of an ongoing Family Medicine peer support group that could continue after they left training

**Table 2:** Skills and attitudes tutors strove to address in the tutorial

Planned Skills and Attitudes to address in tutorials	Session # in which tutors and/or residents noted issue discussed in each Tutorial Group that addressed skill or attitude		
	Group A	Group B	Group C
1. Communication and consultation skills	2,3,7,8	3,4,6	2,6
2. Practicing holistically	2,3,7,8	3	3
3. Data gathering and interpretation	2,4,7,8	2	2
4. Making a diagnosis/decision	2,3,4,7,8	2,4	4,5,9
5. Clinical management	2,3,4,7,8	2,4,6	3
6. Managing medical complexity	7,8	4	-
7. Organizational management and leadership	-	4	-
8. Working with colleagues and in teams	3,7,8,4	-	-
9. Community orientation	4,7,8	4	
10. Maintaining performance, learning and teaching	7,8	-	-
11. Maintaining an ethical approach	7,8	-	-
12. Fitness to practice	-	-	-

- = evidence of skill or attitude not present in the data (tutor and / or resident’s summary notes for session)

### Intervention: online tutorials

Up to eleven Zoom sessions, lasting at least one hour each, were arranged weekly at convenient times for the residents between June through September 2020. Tutors planned the sessions in advance and made adjustments based on the residents’ on-going feedback. Residents were encouraged to bring topics from clinical care to discuss as a group. Due to requests by the residents, the final one to two sessions were dedicated to cognitive behavioral training (CBT).

The usual tutorial started with the assigned resident sharing a real case from their practice with the group, often delivered through role play with a peer, and the tutor and other residents asked questions and gave feedback. Then, the tutor gave a presentation in a case-based, interactive manner. Both tutors and residents reflected on the session afterwards and one resident was assigned to write a session summary and collect evaluations from their peers in the group. Tutors were asked to complete a form as well. (See Additional files 1 and 2 for forms.)

### Data collection

#### Initial data collection at the end of tutorial sessions

During the tutorials, quantitative and qualitative data were collected from available sources [5]. Quantitative data included resident and tutor attendance and participation, numbers of skills and attitudes addressed, and topics covered during the sessions.

A total of 118 documents were available for review and the transcript from an online chat. The number in each category is indicated after data sources: Tutors and residency director meeting minutes (6 documents) were completed at planning and check-in sessions. Tutor tutorial record forms (11 documents) were encouraged, but not mandatory. Resident tutorial evaluation forms (65 documents) and resident session summaries (36 documents) were mandatory and collected by the residency director. One group decided to create a social chat group (WhatsApp) so they could communicate about clinical patients in real-time.

### Data collection two years later

To evaluate the benefit of the program on the residents’ current practice, two years after the tutorials concluded, TZ, AS and LS developed a 16-question survey. Questions were derived from the response content of the first read through of the tutor and resident forms completed after each tutorial. The survey included closed and open questions about: 1) current practice settings and dynamics and current teaching responsibilities; 2) motivation to participate in the tutorials at the time and recollection of the tutorial’s context at the time (safety to learn, participation, tutor’s skills); 3) recalled most and least valuable aspects of the sessions, and elements covered in the tutorials currently being used. (See Additional file 3 for survey questions.) A link was sent via WhatsApp to all participating residents on three occasions, ten days apart in October 2022. Responses were anonymous so we could not identify the responder’s tutor group assignment.

### Analysis

Descriptive frequencies were calculated for demographics and items such as attendance, number of tutorial sessions, etc. Qualitative data were read, reread, and categorized using thematic analysis techniques [7] by TZ, an experienced family physician and qualitative researcher who did not participate in the tutorial program, but was familiar with the residents due to her role as a Fulbright Scholar and visiting professor in the medical department and residency program. The tutor and resident tutorial responses and the resident session summaries were compared with the list of skills and attitudes; sessions in which a skill or attitude was mentioned at least once was noted and coded. Meeting minutes, responses to questions in the forms were read and content was coded and grouped according to topic. Because of the differences in the groups, we also examined each group as an entity.

One resident elected not to attend the mandatory tutorials after the first two sessions; remediation one-to-one sessions were conducted with her and three different tutors so that she could fulfill graduation requirements. These debriefs and evaluations were included with the other data.

The chat text was read by TZ and NS, one of the facilitators of that group. The topical content was added to the qualitative themes. Findings were checked by two additional trainers AS and BC, an Arabic speaker [8]. Survey results are presented separately using descriptive frequencies. Open-ended question responses were read and quotes selected to illustrate repeated observations.

The ANNU IRB provided ethical approval of this study.

## Results

A description of the resident’s age, gender and geographic location in Palestine are presented in Table 3 along with the composition of the three tutorial groups, the number of sessions, and the medical knowledge/clinical cases discussed. We then present the themes and subthemes of the 118 documents described above.

**Table 3:** Resident demographics and composition of the three tutorial groups *n* = 13

Gender: 8 female (62%) 5 male (38%)			
Age: average (range) 35y (34–47)			
Geographic Distribution			
–North: 6			
–Central: 2			
–South: 5			
Tutorial Groups	Group A	B	C
Initial Number of residents	5 = 4 females/1 male	4 = 3 male; 1 female	4 = 2 male; 2 female
Final # of residents	6 females	4 = 3 male	3 = 2 male; 1 female
Tutor demographics	1 F British GP; 1 M US FM Arabic speaker working in Jordan	3 Rotating British GP (2F 1 M), no Arabic	3 British GP M, 1 Arabic speaker familiar with context
Total # Sessions	11	9	8
Timing: Day, Time, Palestine EEST^a^	Weekday 1200	Weekend 1800	Weekday 2100
Resident attendance	Regular	Sporadic	Consistent minus 1
Resident session report	Complete	Complete	Complete
Resident evaluation	Complete	Incomplete	Incomplete
Used Role Play	yes	yes	yes
WhatsApp group	yes	no	no
**Topics discussed during the tutorial sessions**			
	Group A	Group B	Group C
	Fatigue	Fatigue	Fatigue
	Headache	Difficult patient cases	Diabetes
	B12 deficiency/Anemia	Painful diabetic neuropathy	Abdominal pain
	Adverse Drug Reactions	Acne	Breast lump management
	DM patient	Diagnostic reasoning errors	Breaking Bad News
	Managing Angry patients	Insomnia	Urinary incontinence
	Gait abnormalities	Urinary incontinence	Sexual problems
	Dizziness	Recurring abdominal	Heartsink patients^
	Alopecia	pain in children	Knee pain
	Infant with fever		Orthopedic problems
	OSCE Practice	CBT-1 session	CBT-1 session
	Introduction to Cognitive Behavioral therapy (CBT) --2 sessions		

*M* Male gender, *F* Female

Weekend in Palestine is Friday and Saturday. UK is 2 h behind Palestine

^a^East European Summer Time

The major themes of knowledge, skills, attitudes, cultural disconnects and tutorial logistics emerged. All three groups reported the knowledge and skills acquired, with group A listing the most followed by groups B and C. Knowledge gained was related to details learned in case-based discussions such as how to read a complete blood count, obtaining a stool culture on diarrhea due to the high rates of parasite-caused diarrhea in Palestine, and learning how to start insulin. Groups B and C focused on clinical knowledge and tutors noted that it was often difficult to get the residents off the topic of disease management knowledge and onto the educational goals for the program.

Skills acknowledged by the residents fell into the following categories: 1) Consistently using a systematic approach toward a patient which included proficiencies such as taking a thorough illness history, conducting a thorough medication review, performing an exam, inquiring about red flags, remembering to assess psychosocial factors, and safety netting which involved arranging follow up with the patient. 2) The use of evidence-based guidelines to guide decisions included such topics as refraining from ordering unnecessary labs and identifying useful guidelines to reference for diagnoses such as hypertension or fatigue. 3) Good communications skills such as ICE (patient’s ideas, concerns and expectations should be asked), listening well, purposefully asking both open and closed questions, and using silence to allow the patient to feel emotion such as grief. 4) The importance of patient education; and 5) Learning how to give feedback were the final subthemes. Once again, group A residents recorded the most skills in their evaluations followed by B and C. See Table 2, columns 2–4.

Attitudes were frequently noted by the residents in group A and rarely noted in groups B or C. These included the benefit of interaction with colleagues; valuing the experience of the tutors, including the perspectives of other medical cultures (UK and US); seeing every patient as an opportunity to learn; recognizing that not every problem requires a solution; and reflections on professional boundaries and bioethics. Only group A residents mentioned feeling open and honest and willingly shared clinical difficulties during the sessions. One resident in group A even presented her father as a case for the residents to discuss and guide her. Tutors focused on attitudes and skills with the remediating resident including the importance of lifelong learning and team leading skills.

Collegial attitudes likely led the residents in group A to form a social media chat group after the second session so they could share real-time problems they were encountering in clinic. Midway through the program, the residency director assigned the male resident in Group A to another group and added a female resident, who was frustrated by the passivity of the residents in her original group. A review of the chat record showed that the number of messages increased with time. Although the residents were well known to each other, this was the first time that they consulted with one another about practice-related questions on a regular basis. Many guidelines and treatment algorithms were shared between the residents as well as pictures of dermatological cases and a video of a child with a gait problem. The messages showed increasing honesty and vulnerability as well as increased confidence in their clinical expertise. The tutors planned sessions based on the content shared, received immediate feedback about the sessions, and saw evidence of guideline use. Tutors did respond to some questions posed on the chat, but much of the support was from one resident to another. For exemplary text see Table 4.

**Table 4:** Themes and exemplary quotes from Group A’s chat discussions

1. Discussion content enabled the tutors to plan the next session from real time problems residents encountered in their clinics
M*: “We want to talk about differential diagnosis, red flags and management. How to advise parents and how to reassure them as they are always anxious about their sibling.”* T*: “The other case is about girls not walking straight ‘gait problems’.” (also sent a video of the child with limping gait)* M*: “I was choosing a topic of headaches. There is luck here as headache as somewhat connection with anemia and connection in general with fatigue which has been discussed with us in previous tutorial.”* R*: “I have two case discussion that I would like to share with you this Saturday. The first one is about a patient 8 year old coming with mild gastroenteritis*
2. Building trust and relationships within the group
Continuing the above text from R about the 8 year old: “*His mother asked for CBC (complete blood count). On physical exam patient was well, not pale. I asked the mother why she wants me to do a CBC she said because her son feels tired these days. I was against repeating CBC and told the mother that she needs to give him ORS and come again in two weeks for follow up and re-evaluate his tiredness. Mom came back in two weeks bringing her youngest daughter 6 months for vaccination but was referred to me for evaluation as she had bronchiolitis. When I started examining her daughter she told me in an angry way:* *‘Do you remember when I asked you to do a CBC for my son and you refused !! I went to another doctor who did the CBC and he saw amoeba in his CBC.’”* R posed a question to the group: “*What do you think so far about what happened and what to do next? PS: unfortunately I become upset.”*
3. Sharing feelings openly in the group is also demonstrated in the above quote. Another example about a case seen after an earlier session included discussion of the topic:
T: “*Good morning. Today I deal with another case of alopecia areata but now I was more comfortable and I felt with trust. Thanks our teachers.”*
4. Seeking help and support from their peers became more frequent as the trust and relationships grew in the group and they started using their skills mix and asking for support from each other
Having discussed how to manage her father with poorly controlled blood pressure, polypharmacy and diabetes mellitus with the group in a tutorial session, M then sought the help from L, a fellow resident in the group who ran a diabetes clinic M: *“Good evening. Today my dad started basal insulin. He was so afraid of the needle. I brought him to the clinic and me and L gave him the injection and showed him how to take it. It was easy. He was happy that there is no pain with the needle. I will follow him closely* *These sessions help me to organize my ideas in a systematic way. Gave me the courage to take decisions with confidence. And simplify the cases which I thought was hard. Thanks a lot.”*
5. Evidence of the use of and interest in guidelines and patient education leaflets
M: “*Thanks a lot for your effort everybody. Can we have a NICE guideline for anemia and fatigue. In order to print it and have it with us at the clinics*.” T shared a complicated case about a fever and concluded: “*I send to her Arabic brochures about dealing with high fever*.”
6. Feedback on the tutorial sessions
T: *“The most important thing that changed is my personality. I was Very nervous when I deal with angry patients. Now I learned patience. Also I learned from the last session the case of M's dad, some art of medicine, how to deal with polypharmacy. I printed some of the important schemes for diagnosis and management diseases. Really we are lucky to join this group. Thanks a lot.”* L: *“Good evening. Me and R will be on duty this Saturday…what about our session, we don't like to miss it.”*

Groups B and C were less collegial and comments to the Resident Tutorial Evaluation question: “What do I still need to learn?” more commonly showed answers such as “From my side …. nothing” or “as a fourth year resident I don’t need this.”

Group B and C covered fewer of the recommended skills and attitudes (Table 2) and the tutors in Group C rarely turned in a tutorial debrief. Group C residents showed less reflection in their session evaluations.

### Cultural disconnects

 Evaluation reports and the residency director’s notes from check-ins with the residents showed trouble with a tutor speaking English too quickly, residents offended by the humor of one UK tutor, and nuances during case presentations and discussions that were misunderstood without an Arabic tutor present. A lack of understanding of the Palestinian context and the types of cases encountered caused discussions to get bogged down in disease management instead of the approach to the patient and the process of thinking through care decisions that reflected a more patient-centered approach.

### Logistics lessons learned

Role plays were done by all groups and seemed to work the best if the residents did the role play in Arabic, with an Arabic tutor present. Role plays done by the tutors or performed in English by the residents occurred in several cases, and were deemed less effective. Tutors who asked residents to prepare for sessions and to bring cases from their clinical work, and were well-prepared themselves, had better resident participation in sessions. Tutor pair instability due to the voluntary nature of the role, and busy clinical schedules of their own, resulted in less organized sessions. PowerPoint used with discussions was initially tried and then avoided because discussions were easier to manage when all faces were visible with a second tutor managing the chat function.

Both the tutors and residents experienced a learning curve with the Zoom platform. Weak home internet systems often caused problems such as needing to turn off cameras or residents needing to sign on several times and missing parts of the session. Erratic electricity due to regional cuts was also a challenge. One resident noted on their evaluation: “I am very lucky because the electricity was on.” Lockdowns due to COVID-19 quarantines and needing to be on medical duty sometimes interrupted attendance. Although residents were assigned to times that were agreeable to them, interruptions still occurred due to family obligations and background noise from children and music.

### Survey results

The follow-up survey two years later had a 77% response rate (10/13). We suspect that the three who did not respond did not find the tutorial particularly helpful and were the less active members in their groups. Nine of the responders were in clinical practice, one also had a private clinic, and one served an administrative role in government. In addition to seeing patients, one supervised residents, one taught courses at the medical school, and two conducted research. Graduates doing patient care (9/10) in family health centers were able to use their Family Medicine skills most of the time. Too many patients to see, followed by unsupportive administrations and colleagues were the main barriers for using their Family Medicine skills.

Most reported attending all the tutorial sessions, were motivated to participate, and would have done so if it wasn’t obligatory (7/10). All felt the tutors created a safe learning environment and most (9/10) felt the manner of teaching was useful. Two thirds (6/10) thought their colleagues actively participated and three did not, with one interpreting a colleague’s attitude as “thinking the tutorials were a joke.” Another was aware that a peer signed on to Zoom but was not listening. Barriers to participation included: unstable internet (4) and other obligations at home (6), despite confirming the best times beforehand. Two found it hard to understand the tutors, two thought the cases didn’t relate to the patients they saw, and one felt the covered topics were unhelpful. The most valuable parts of the tutorial were help in organizing thinking (5), networking with colleagues (4), learning the UK perspective (3), the chat group (3), and increased confidence in patient care decisions (3). Most (7/9) in clinical practice used decision support such as Epocrates and the American Family Physician.

## Discussion

This case study evaluates the facilitators and hinderances of a 2020 transnational online tutorial program to enhance practice readiness skills for 13 FM residents preparing to practice in Palestine, a low/middle income country [9]. The highest functioning group had the most stable tutor pair who stayed focused on the learning goals, included one Arabic speaker who was familiar with the context, and fostered trusting collegial relationships that led to the learners taking the initiative to create a chat group for real-time communication about patient care. The membership evolved into all female learners. Two years later, most learners (9/13) reported the tutorials were useful and employed learnings like the use of decision supports in their practices, but all struggled with fully implementing what they learned due to the structural challenges in their practice settings. Some residents reported consulting each other about difficult cases, but the chat group had dissolved.

While virtual learning occurred prior to the pandemic [1–4], the pandemic increased the variety of simulation, social media, and online methods in both developed and developing countries to enhance student and postgraduate health education [10, 11]. MacNeill writes, "Higher education experts agree that by 2025, only a minority of learners will be either fully face two face or fully online, with the majority attending in blended and HyFlex formats" [10]. One review identified 77 systematic reviews that evaluated the effectiveness of digital education interventions across several health care disciplines, of which 79% were performed in high-income countries [12]. Specifically noted in the conclusion of this study was the need for such studies from low and middle-income countries.

This case study, in a low/middle income country, examined the virtual training of residents to improve clinical decision making and enhance patient centered care. The effort attempted to fill in for inadequate supervision in the clinical setting. Our training was different than delivering medical education to students [2], continuing education to doctors, or skills like life support which were reported in other studies in Palestine [3]. To our knowledge, no studies have been performed to evaluate the effectiveness of transnational tutorials to enhance the clinical decision making training of residents. While medical education in Palestine requires some fluency in English, most patient care and clinical discussions occur in Arabic. Hence, the presence of an Arabic speaker was essential to help with the nuances of case discussions, such as how to manage an angry patient, break bad news, or how to adapt Western-model communication skills in a culturally appropriate manner. Familiarity with the cultural context was essential to understand leaning styles and choose cases and management options appropriate to what the learners were seeing clinically. Avoiding the judgment often accompanying people from high income countries while training others in low income countries was also very important. Trainers needed to understand, validate and appreciate the cross cultural context in which they were teaching [13]. This is in agreement with cross cultural training guidelines [14].

The extreme focus on clinical knowledge by some residents was partly cultural in that they had limited experience with a problem-based, case-based style of learning [15]. They felt safer demonstrating their clinical knowledge training even if it was unrelated to the issue being discussed and less comfortable discussing what they did not know in front of their tutors and peers. In a culture where honor and shame are high ideals, it can be considered rude to give someone anything but positive feedback in front of others [16]. Some tutors first modeled constructive feedback in their efforts to create a non-judgmental and safe environment and to help foster self-reflective learning.

Creating a safe environment was especially important in allowing learners to ask questions and show what they did not know. Hence, one of the important factors to promote learning in the virtual environment identified by Hovlid’s focus group study with students in Norway was present [17]. While all residents responding to the survey reported a safe learning environment, the group with all female learners, a female tutor and a male tutor who spoke Arabic and was familiar with the context seemed to create a level of safety and trust that surpassed the other groups. This finding illustrated the importance of being able to address language and cultural differences when giving training focused on decision making and communication skills in the clinical setting, especially when working with others from a different culture and background. The degree in which gender dynamics among residents played a role in group effectiveness is unclear and would be an interesting topic for further study.

Intrinsic and extrinsic barriers affected the success of the program; both are difficult to address but should be considered. Some of the residents were open to new ideas and saw the value of the sessions. Because the residency director and head tutor worried that some residents did not have enough insight into their own learning needs to realize that they needed to be exposed to this approach to learning, the sessions were made compulsory. This lack of internal motivation is an intrinsic barrier. In contrast, the group with all female learners that developed the social chat did this themselves to further enhance their learning without any suggestion by the tutors, demonstrating the value of intrinsic motivation. Chinese medical providers illustrated the same value of intrinsic motivation by self-initiating a social media platform to overcome geographic barriers and to improve communication between healthcare professionals, enhance collaboration, and provide increased access to up-to-date medical guidelines [18].

Extrinsic barriers, reported by others in Palestine [2, 3], included low internet bandwidth, and power outages. These were impossible to manage and needed to be planned for by taping the sessions. Finally, the lack of continuity in the Palestinian clinical system made it difficult to provide patient specific feedback that the residents could immediately implement since the resident may not see the same patient in the future. This reality stands in direct opposition to one of the core concepts of FM which is continuity of care [19]. Even during the tutorial, it was clear that system changes would need to occur for the FM residents to fully implement their training. The follow-up survey demonstrated only a little progress. 

### Limitations

The limitations to this evaluation include noncompliance with our data collection efforts despite our prodding. Some tutors and residents did not complete evaluation forms and reflections during the tutorial, and three residents did not participate in the follow-up survey two years later. Recall about the tutorials may be inaccurate, but information on how they were applying what they had learned in their current practice settings and the challenges of their clinical sites are valuable for future planning. We attempted to collect data from multiple sources to provide as complete and honest a representation as possible. The head researcher did not participate in the tutorials, with the intention of limiting bias. Her conclusions were checked by several tutors. Despite these difficulties, the information still provides important knowledge about the educational processes in a region that the mainstream literature overlooked.

This study examines the facilitators and inhibitors of a transnational tutorial run during the pandemic to improve readiness to practice skills. Given the transnational nature of the tutorial, UK and US tutors and Palestinian residents, stable tutor pairs that included an Arabic speaker familiar with the context fostered safety and encouraged the trust and openness to address cultural challenges when they became aware of them. Intrinsic barriers among learners such as not perceiving a need for the program and external barriers such as internet dependability, and practice structures that do not support core elements of family medicine are more difficult to address and require planning and changes at the health system level in Palestine.

## Conclusion

Tutorials focused on clinical thinking and decision-making skills were most successful when the tutorial pair was stable, offered familiarity with the language and addressed cultural differences. Intrinsic factors such as lacking the motivation to participate and extrinsic factors such as unstable internet and rolling electric cuts, and clinical structures that made applying new skills challenging were more difficult to address but also must be considered.

### Supplementary Information

**Additional file 1. **Tutor Tutorial Record Form to be completed after each session.

**Additional file 2. **Resident Tutorial Evaluation form to be completed after each tutorial.

**Additional file 3. **Online Follow Up Survey Questions.

## Data Availability

The datasets used and/or analyzed during the current study are available from the corresponding author on reasonable request.

## Abbreviations

NGO: Non-governmental organization
IRB: Internal review board
ANNU: An-Najah National University
GP: General Practitioner
MOH: Ministry of Health
UK: United Kingdom
US: United States of America
ICE: Ideas, Concerns, Expectations
CBT: Cognitive Behavioral Therapy
FFMP: Foundation for Family Medicine in Palestine
MAP: Medical Aid for Palestinians
FM: Family Medicine
