# Supplementary material for: A case study: lessons learned from online tutorial to improve practice readiness for family medicine residents in Palestine
Source: BMC Med Educ. 2024 Mar 8;24:262. doi: 10.1186/s12909-024-05163-1 (PMC10924311; doi:10.1186/s12909-024-05163-1)
Supplement: Supplementary file 1 — Additional file 1. Tutor Tutorial Record Form to be completed after each session. [file 12909_2024_5163_MOESM1_ESM.docx]

**APPENDIX 1:** **Tutor Tutorial Record Form to be completed after each session.**

If you can find time to fill this in after each tutorial, I will collate the feedback to share any important learning points with the rest of the tutors, faculty at An Najah and FFMP

Please feedback on the following areas: Attendance, IT issues, timekeeping issues, educational issues e.g. learning methods that worked well, learning needs identified, issues to be covered in future tutorials,

Name of tutor(s):

Group number:

Date:

Topic:

Specific areas focused on

1. Communication and consultation skills
2. Practicing holistically
3. Data gathering and interpretation
4. Making a diagnosis/decisions
5. Clinical management
6. Managing medical complexity
7. Organizational management and leadership
8. Working with colleagues and in teams
9. Community orientation
10. Maintaining performance, learning and teaching
11. Maintaining an ethical approach
12. Fitness to practice

Want went well?

Any problems?

Any ideas to incorporate into further tutorials?/change practice in the future

Any suggestions for the program overall?
