# Supplementary material for: A case study: lessons learned from online tutorial to improve practice readiness for family medicine residents in Palestine
Source: BMC Med Educ. 2024 Mar 8;24:262. doi: 10.1186/s12909-024-05163-1 (PMC10924311; doi:10.1186/s12909-024-05163-1)
Supplement: Supplementary file 2 — Additional file 2. Resident Tutorial Evaluation form to be completed after each tutorial. [file 12909_2024_5163_MOESM2_ESM.docx]

**APPENDIX 2**: **Resident Tutorial Evaluation form to be completed after each tutorial**

Tutorial record sheet

Name:

Date:

Topic:

What 3 things did I learn?

1.

2.

3.

How will I change my practice?

What do I still need to learn?

What did you like about the tutorial?

Any suggestions for how the tutorial can be improved?
