# Supplementary material for: A case study: lessons learned from online tutorial to improve practice readiness for family medicine residents in Palestine
Source: BMC Med Educ. 2024 Mar 8;24:262. doi: 10.1186/s12909-024-05163-1 (PMC10924311; doi:10.1186/s12909-024-05163-1)
Supplement: Supplementary file 3 — Additional file 3. Online Follow Up Survey Questions. [file 12909_2024_5163_MOESM3_ESM.docx]

**APPENDIX 3:** **Online Follow Up Survey Questions**

ANNU Family Medicine is trying to improve the format of future online tutorials. Please answer questions in this survey honestly. Your answers will not be linked to your name and will not affect your future interactions with ANNU.

1. What are your current clinical and teaching settings? (Check all that apply)

- MOH GP clinic
- MOH FM clinic
- Private FM clinic
- Teach FM courses at university
- Conduct research with university
- Clinical supervisor of residents
- Supervise students in the clinical setting
- Administrative work

2. What percent of the time are you able to practice family medicine in your clinical setting?

- MOH 0-100%
- Private 0-100%
- Other ____

3. What prevents you from practicing Family Medicine most of the time in clinical practice?

(Check all that apply)

- Too many patients
- Clinic staff not oriented to Family Medicine approach
- Medical colleagues not supportive of FM approach
- Administration not supportive of FM practice
- Other----
- Not applicable

4. What areas of family medicine do you feel that you need more education?

(Check all that apply)

- Geriatric patient
- Mental health care
- Approach to complex patient
- Women’s health
- Adolescent health
- Other----

5. Thinking back to the clinical tutorials offered by FFMP/MAP to help you prepare for your boards during COVID-19, how motivated were you to participate in the tutorials?

0-100%

6. What percent of the tutorial sessions did you participate in?

0-100%

7. What prevented you from participating? (Check all that apply)

- Bad internet connection
- Obligations at home during the scheduled time.
- Other priorities
- Didn’t think the topics covered helped much
- Cases didn’t relate to the patients I see
- Hard to understand the tutors language
- Other

8. Did the tutors create a safe learning environment for you?

y/n—why

9. Did you feel like your colleagues actively participated in the session

y/n—why

10. Did you have the same tutor(s) for each session?

y/n

11. Did the tutor teach you in a way that was useful to you?

y/n—why

12. What were the most valuable part of the tutorials for you? (Check all that apply)

- Networking with colleagues during the tutorial
- Communicating with colleagues at other times through WhatsApp
- Arabic speaking faculty
- Learning UK perspective
- Learning go-to resources for clinic questions
- Organizing my thinking and approach to patients
- Feeling more confident in making decisions about patient care
- Other___

13. What was the least valuable part of the tutorials for you? (Check all that apply)

- Language barrier
- Scheduled at a difficult time
- Tutorial session lasted too long
- Dynamics of the session did not help me learn

14. If attendance was not obligatory, would you have attended

y/n

15. What guidelines / books / internet support do you use in your daily practice to ensure you are prescribing medications safely and not causing potentially serious interactions or side effects.

____________

• I do / do not use a prescribing support

16. What clinical based evidence do you go to now?

- NICE guidelines
- American Family Physician
- Medscape
- Center for Disease control
- Cochrane reviews
- WHO
- Other ---

Other comments __________
